# A Genome-Wide Association Study Reveals a Rich Genetic Architecture of Flour Color-Related Traits in Bread Wheat

**DOI:** 10.3389/fpls.2018.01136

**Published:** 2018-08-03

**Authors:** Shengnan Zhai, Jindong Liu, Dengan Xu, Weie Wen, Jun Yan, Pingzhi Zhang, Yingxiu Wan, Shuanghe Cao, Yuanfeng Hao, Xianchun Xia, Wujun Ma, Zhonghu He

**Affiliations:** ^1^Crop Research Institute, National Engineering Laboratory for Wheat and Maize, Key Laboratory of Wheat Biology and Genetic Improvement in the Northern Yellow-Huai Rivers Valley of Ministry of Agriculture, Shandong Academy of Agricultural Sciences, Jinan, China; ^2^National Wheat Improvement Center, Institute of Crop Sciences, Chinese Academy of Agricultural Sciences, Beijing, China; ^3^Institute of Cotton Research, Chinese Academy of Agricultural Sciences, Anyang, China; ^4^Crop Research Institute, Anhui Academy of Agricultural Sciences, Hefei, China; ^5^School of Veterinary and Life Sciences, Murdoch University and Australian Export Grains Innovation Centre, Perth, WA, Australia; ^6^International Maize and Wheat Improvement Center, Beijing, China

**Keywords:** brightness (L^*^), candidate gene, GWAS, redness (a^*^), yellowness (b^*^), yellow pigment content (YPC)

## Abstract

Flour color-related traits, including brightness (L^*^), redness (a^*^), yellowness (b^*^) and yellow pigment content (YPC), are very important for end-use quality of wheat. Uncovering the genetic architecture of these traits is necessary for improving wheat quality by marker-assisted selection (MAS). In the present study, a genome-wide association study (GWAS) was performed on a collection of 166 bread wheat cultivars to better understand the genetic architecture of flour color-related traits using the wheat 90 and 660 K SNP arrays, and 10 allele-specific markers for known genes influencing these traits. Fifteen, 28, 25, and 32 marker–trait associations (MTAs) for L^*^, a^*^, b^*^, and YPC, respectively, were detected, explaining 6.5–20.9% phenotypic variation. Seventy-eight loci were consistent across all four environments. Compared with previous studies, *Psy-A1, Psy-B1, Pinb-D1*, and the 1B•1R translocation controlling flour color-related traits were confirmed, and four loci were novel. Two and 11 loci explained much more phenotypic variation of a^*^ and YPC than phytoene synthase 1 gene (*Psy1*), respectively. Sixteen candidate genes were predicted based on biochemical information and bioinformatics analyses, mainly related to carotenoid biosynthesis and degradation, terpenoid backbone biosynthesis and glycolysis/gluconeogenesis. The results largely enrich our knowledge of the genetic basis of flour color-related traits in bread wheat and provide valuable markers for wheat quality improvement. The study also indicated that GWAS was a powerful strategy for dissecting flour color-related traits and identifying candidate genes based on diverse genotypes and high-throughput SNP arrays.

## Introduction

Bread wheat (*Triticum aestivum* L.) is among the most important food crops and is one of the most traded commodities in world markets (Curtis and Halford, 2014). Developing cultivars with appropriate end-use quality is the primary objective of all wheat breeding programs. Flour color plays a significant role in the end-use quality of wheat, particularly for Asian noodles and steamed bread, since it affects consumer acceptance, market value and human nutrition (Zhai et al., 2016a). Color measurements were expressed as tristimulus parameters (L^*^, a^*^, and b^*^). L^*^ is a measure of flour brightness, ranging from 0 (black) to 100 (white); a^*^ measures redness when positive or greenness when negative; and b^*^ describes the yellow-blue color value, and is positive for yellowness and negative for blueness (Hutchings, 1999). Yellow pigment content (YPC) is the most important determinant of flour yellowness caused mainly by accumulation of carotenoids in the grain (Mares and Campbell, 2001). It is also a very important quality criterion for pasta made from durum wheat. Understanding the genetic basis of flour color-related traits (L^*^, a^*^, b^*^, and YPC) is necessary for improving wheat quality by marker-assisted selection (MAS).

During recent decades, numerous quantitative trait loci (QTL) for flour color-related traits have been identified using bi-parental populations (Mares and Campbell, 2001; Patil et al., 2008; Zhang and Dubcovsky, 2008; Zhang et al., 2009; Blanco et al., 2011; Roncallo et al., 2012; Crawford and Francki, 2013a; Colasuonno et al., 2014; Zhai et al., 2016b). Cloning genes relevant to flour color and developing functional markers have become major research focus (Mares and Campbell, 2001; He et al., 2008, 2009; Howitt et al., 2009; Zhang et al., 2011; Crawford and Francki, 2013b). As observed in previous studies flour color is a complex trait, and knowledge of the genetic control is still limited due to use of low density marker platforms and low resolution in bi-parental mapping studies. Phytoene synthase 1 (PSY1) catalyzes the first committed step in carotenoid biosynthesis, and it is generally accepted as the most important regulatory node, significantly correlated with carotenoid accumulation and determined flour color (*r* = 0.8) (Zhai et al., 2016c). Thus, more effort is needed to further dissect the genetics of flour color-related traits.

Genome-wide association study (GWAS), based on linkage disequilibrium (LD), is an effective complementary strategy to QTL mapping in dissecting associations between genotype and phenotype in germplasm collections (Yu and Buckler, 2006; Yu et al., 2006; Zhu et al., 2008). GWAS has a number of advantages over traditional linkage mapping, including the use of germplasm populations, potential for increased QTL resolution, and a wide sampling of molecular variation (Buckler and Thornsberry, 2002; Flint-Garcia et al., 2005; Waugh et al., 2009). With the development of high-throughput genotyping platforms, GWAS has been increasingly used to identify loci responsible for complex traits in plants, including wheat (Brachi et al., 2010; Zhao et al., 2011; Wang et al., 2012; Marcotuli et al., 2015; Tadesse et al., 2015). A potential disadvantage is identification of spurious associations. The mixed linear model (MLM) method is an effective method to avoid spurious associations as it simultaneously accounts for population structure (Q) and genetic relatedness (K) between individuals (Yu and Buckler, 2006; Yu et al., 2006). The wheat Illumina 90 K SNP array made a dramatic improvement in the number of gene-based markers, and widely used to detect QTL for important traits and identify candidate genes (Ain et al., 2015; Sun et al., 2017). Nevertheless, the genetic distances between markers were large on wheat chromosomes using the 90 K SNP array, particularly a low coverage in the D genome, reducing the power of marker identification and the precision of QTL mapping (Liu et al., 2017). Recently, the wheat Axiom 660 K SNP array was developed, providing higher marker density, higher resolution and better coverage of wheat genome (Cui et al., 2017).

The objectives of the present study were to: (1) identify the genetic basis underlying flour color-related traits in a collection of 166 bread wheat accessions using the wheat 90 and 660 K SNP arrays, and 10 allele-specific markers for known relevant genes; and (2) propose candidate genes affecting flour color-related traits based on biochemical information and bioinformatics analyses, with the ultimate aim of facilitating molecular breeding. Finally, the genetic determinants of flour color-related traits could be identified more accurately by GWAS using diverse genotypes and high-throughput SNP arrays.

## Materials and methods

### Plant material and field trials

The association panel used in this study was a genetically diverse collection, comprising 166 elite bread wheat cultivars mainly from Yellow and Huai Valley of China, but also Italy, Argentina, Japan, Australia, and Turkey. Information about the accessions, including cultivar names, origins and subpopulation identity, was described in Table S1.

Briefly, the materials were grown in the 2012–2013 and 2013–2014 in randomized complete blocks with three replications at Anyang (AY, Henan province, 35°12′N, 113°37′E) and Suixi (SX, Anhui province, 33°17′N, 116°23′E), providing data for four environments. Each plot comprised three 2 m rows in 2012–2013 and four rows in 2013–2014, with 20 cm between rows. Agronomic management was performed according to local practices.

### Phenotypic evaluation and statistical analysis

For phenotypic evaluation 500 g clean grain from each plot was ground in a Brabender Quadrumat Junior Mill (Brabender Inc., Duisberg, Germany; http://www.cwbrabender.com) with a 0.15 mm sieve. Kernel hardness was determined with a Single Kernel Characterization System (SKCS 4100, Perten, Sweden). Moisture and protein contents were measured using a Near Infrared Transmittance (NIT; Foss-Tecator 1241, Foss, Högänas, Sweden). The data of kernel hardness, moisture and protein content were only used to calculate how much water is required to tempering, so we did not describe these in this manuscript. Before milling, samples were conditioned to 14, 15, and 16% moisture overnight for soft (SKCS hardness index, HI < 40), medium (HI, 40–59), and hard (HI > 60) types, respectively, as mentioned by Jin et al. (2016). Due to time constraints and a laborious milling method only two replications were made for each environment.

Flour color parameters [brightness (L^*^), redness (a^*^) and yellowness (b^*^)] were measured with a Minolta colorimeter (CR-310, Minolta Camera Co., Ltd., Osaka, Japan) using the Commission Internationale de l'Éclairage (CIE) L^*^ a^*^ b^*^ color system, respectively (Oliver et al., 1992). YPC was assessed according to Zhai et al. (2016b). Briefly, 1 g of flour sample was extracted with 5 ml of water-saturated n-butanol, along with shaking on an orbital incubator for 1 h at room temperature. After centrifuging at 5,000 rpm for 10 min, the absorbance was measured at 436.5 nm. YPC was expressed as μg.g^−1^ using a correction coefficient of 0.301 (American Association for Cereal Chemistry, 2000). Each sample was assayed twice, and a third assay was performed if the difference between two repeats was more than 10%. The mean values were used for statistical analysis.

Statistical analysis was carried out using SAS 9.2 software (SAS Institute, Inc., Cary, NC, USA, http://www.sas.com). Briefly, basic statistics such as means, standard deviation, and minimum and maximum values were calculated with the UNIVARIATE procedure. Analysis of variance was performed using the PROC MIXED procedure, where environments were treated as fixed effects, and genotypes, genotype × environment interaction and replicates nested in environments as random effects. Broad-sense heritability (*h*^2^) was calculated following Zhai et al. (2016b). For each trait, a best linear unbiased predictor (BLUP) for each accession of each trait was calculated across four environments and used for further analyses. Pearson's correlation coefficient (*r*) among the flour color-related traits was calculated using the PROC CORR procedure based on BLUP values.

### Genotypic characterization

Genomic DNA was extracted from seedling leaves using the CTAB (cetyltrimethylammonium bromide) method (Doyle and Doyle, 1987). All 166 accessions were genotyped by both the Illumina wheat 90 K (comprising 81,587 SNPs) and Affymetrix wheat 660 K (containing 630,517 SNPs) SNP arrays by Capital Bio Corporation, Beijing, China (http://www.capitalbiotech.com/). SNP allele clustering and genotype calling were performed using the polyploid version of GenomeStudio software (Illumina, http://www.illumina.com). The default clustering algorithm was initially used to classify each SNP call into three allele clusters. Manual curation was then performed for more accurate genotyping. Markers with a minor allele frequency (MAF) < 5%, and more than 20% missing data were removed from the data matrix. After stringent filtration 259,922 SNP markers from the wheat 90 and 660 K SNP arrays were integrated into a common physical map for GWAS. The physical positions of SNP markers were obtained from the International Wheat Genome Sequencing Consortium (IWGSC RefSeq v1.0, http://www.wheatgenome.org/) and used in association analysis.

Given that many of the genes influencing flour color-related traits have been well characterized, allele-specific markers for *Psy-A1* (He et al., 2008), *Psy-B1* (He et al., 2009), *TaPds-B1* (Dong, 2011), *e-Lcy3A* (Crawford and Francki, 2013b), *Talcye-B1* (Dong, 2011), *Lox-B1* (Geng et al., 2012), *Pinb-D1* (Giroux and Morris, 1997), and 1B•1R translocation (Liu et al., 2008) were used to genotype the association panel following the indications reported in the previous works to assess whether marker–trait associations (MTAs) identified in this study were co-located with these known genes. Detailed information of these markers is provided in Table S2.

### Population structure and linkage disequilibrium analysis

A subset of 2,000 polymorphic SNP markers, distributed evenly across all the wheat chromosomes, was selected for population structure analysis using STRUCTURE (Pritchard et al., 2000). We applied the admixture model with correlated allele frequencies to assess numbers of hypothetical subpopulations ranging from *K* = 2 to 12 using 10,000 burn-in iterations followed by 100,000 MCMC (Markov Chain Monte Carlo) replicates. For each *K*, five independent runs were carried out. According to Evanno et al. (2005), the 166 accessions were structured into three subpopulations as described in Liu et al. (2017).

Pairwise LD was calculated as squared correlation coefficients (*r*^2^) among alleles, and significance was computed by 1,000 permutations using TASSEL (Bradbury et al., 2007). LD was calculated separately for loci on the same chromosome (intra-chromosomal pairs) and unlinked loci (inter-chromosomal pairs). The extent and distribution of LD were graphically displayed by plotting intra-chromosomal *r*^2^-values for loci in significant LD at *P* ≤ 0.001 against the physical distance, and a locally weighed polynomial regression (LOESS) curve was fitted using XLSTAT (Addinsoft, Paris, France). The critical *r*^2^-values beyond which LD is due to true physical linkage was calculated by taking the 95th percentile of the square root transformed *r*^2^-values of unlinked markers (Breseghello and Sorrells, 2006). The intersection of the LOESS curve with the line of the critical *r*^2^ was estimated to see how fast LD decay occurs.

### Marker-trait association analysis

Marker-trait association analysis was performed separately for each environment and BLUP values across four environments using the MLM in TASSEL. Association mapping model evaluations were based on visual observations of the quantile-quantile (Q-Q) plots, which are the plots of observed –log_10_ (*P-*value) vs. expected –log_10_ (*P-*value) under the null hypothesis that there was no association between marker and phenotype. A false discovery rate (FDR) of 0.05 was used as a threshold for significant association (Benjamini and Hochberg, 1995). The association of a marker with a trait was represented by its *R*^2^-value, an estimate of the percentage of variance explained by the marker. To provide a complimentary summary of declared putative MTAs, Manhattan plots were generated using a script written in R software (http://www.r-project.org/).

To assess the pyramiding effect of favorable alleles of MTAs for flour color-related traits identified in this study, the BLUP value for each trait in each accession was regressed against the number of favorable alleles using the line chart function in Microsoft Excel 2016.

### Prediction of candidate genes for flour color-related traits

To assign putative biological functions of significant SNP markers associated with flour color-related traits, the flanking sequences of SNPs were blasted against the NCBI (http://www.ncbi.nlm.nih.gov/), European Molecular Biology Laboratory (http://www.ebi.ac.uk/) and European Nucleotide Archive (http://www.ebi.ac.uk/ena) public databases following Zhai et al. (2016b).

In addition, chromosomal locations and physical distances of 40 known genes influencing flour color-related traits, mainly involved in terpenoid backbone biosynthesis and carotenoid biosynthesis and degradation, were evaluated using IWGSC RefSeq v1.0. If the physical distances between MTAs identified in this study and known genes were less than LD decay, the MTAs were considered to be co-located with these known genes. Following Colasuonno et al. (2017), these 40 known genes were blasted against the available dataset of SNP marker sequences (Wang et al., 2014). Markers aligned with more than 80% similarity were considered to be within the coding sequences of known genes. Gene ontology annotation for the candidate genes was also conducted using EnsemblePlants (http://plants.ensembl.org/index.html).

## Results

### Phenotypic variation

There was a wide range of phenotypic variation for flour color-related traits among the 166 accessions, particularly for a^*^, b^*^ and YPC where nearly 3 to 16-fold differences were observed (Table 1). Across all four environments, the mean L^*^ was 90.29, ranging from 87.29 to 92.06; a^*^ averaged −0.86, ranging from −1.75 to −0.11; b^*^ varied from 5.29 to 14.56 and averaged 8.83; and YPC averaged 1.18 μg.g^−1^, with a range of 0.58–2.95 μg.g^−1^. The frequency distribution of each trait in each environment is provided in Figure S1.

**Table 1:** Phenotypic variation of flour color-related traits in 166 bread wheat cultivars across four environments.

**Trait**	**Mean**	**SD**	**Min**	**Max**	***h^2^***
L^*^	90.29	0.92	87.29	92.06	0.89
a^*^	−0.86	0.34	−1.75	−0.11	0.91
b^*^	8.83	2.00	5.29	14.56	0.92
YPC	1.18	0.42	0.58	2.95	0.93

L^*^, flour brightness; a^*^, flour redness; b^*^, flour yellowness; YPC, yellow pigment content (μg.g^−1^).

*SD, standard deviation*.

*h^2^, broad-sense heritability*.

High broad-sense heritabilities (*h*^2^ ≥0.89) were observed for these traits, indicating that genetic effects played a determinant role for each trait (Table 1). Analysis of variance indicated that the effects of genotypes, environments, and their interactions were significant for flour color-related traits, and genotype had a larger effect (Table S3). Pearson's correlation coefficients among all flour color-related traits were mostly significant (*P* < 0.001), except for L^*^ × a^*^ (Table S4). b^*^ was negatively correlated with L^*^ (*r* = −0.71) and a^*^ (*r* = −0.68). YPC was negatively correlated with L^*^ (*r* = −0.28) and a^*^ (*r* = −0.89), and positively correlated with b^*^ (*r* = 0.83).

### Population structure and linkage disequilibrium

According to Evanno et al. (2005), Δ*K* was plotted against the number of subgroups K. The maximum value of Δ*K* occurred at *K* = 3, indicating that a *K*-value of 3 was the most probable prediction for the number of subpopulations (Liu et al. 2017). In general, subgroup 1 comprised 62 cultivars primarily derived from Shandong province and abroad; subgroup 2 consisted of 54 varieties predominantly from Henan, Shaanxi and Anhui provinces; and subgroup 3 included 50 cultivars mainly from Henan and Hebei provinces.

For the whole genome the threshold *r*^2^, calculated as the 95th percentile of the distribution of *r*^2^ of unlinked markers, was 0.082, and thus all values of *r*^2^ > 0.082 were probably due to physical linkage. The intersection between the threshold *r*^2^ and the LOESS curve was at 8 Mb, which was considered as the LD decay rate of the population. Similarly, the LD decay was 6, 4 and 11 Mb for A, B and D sub-genomes, respectively.

### Marker-trait association

**Figure 1:**
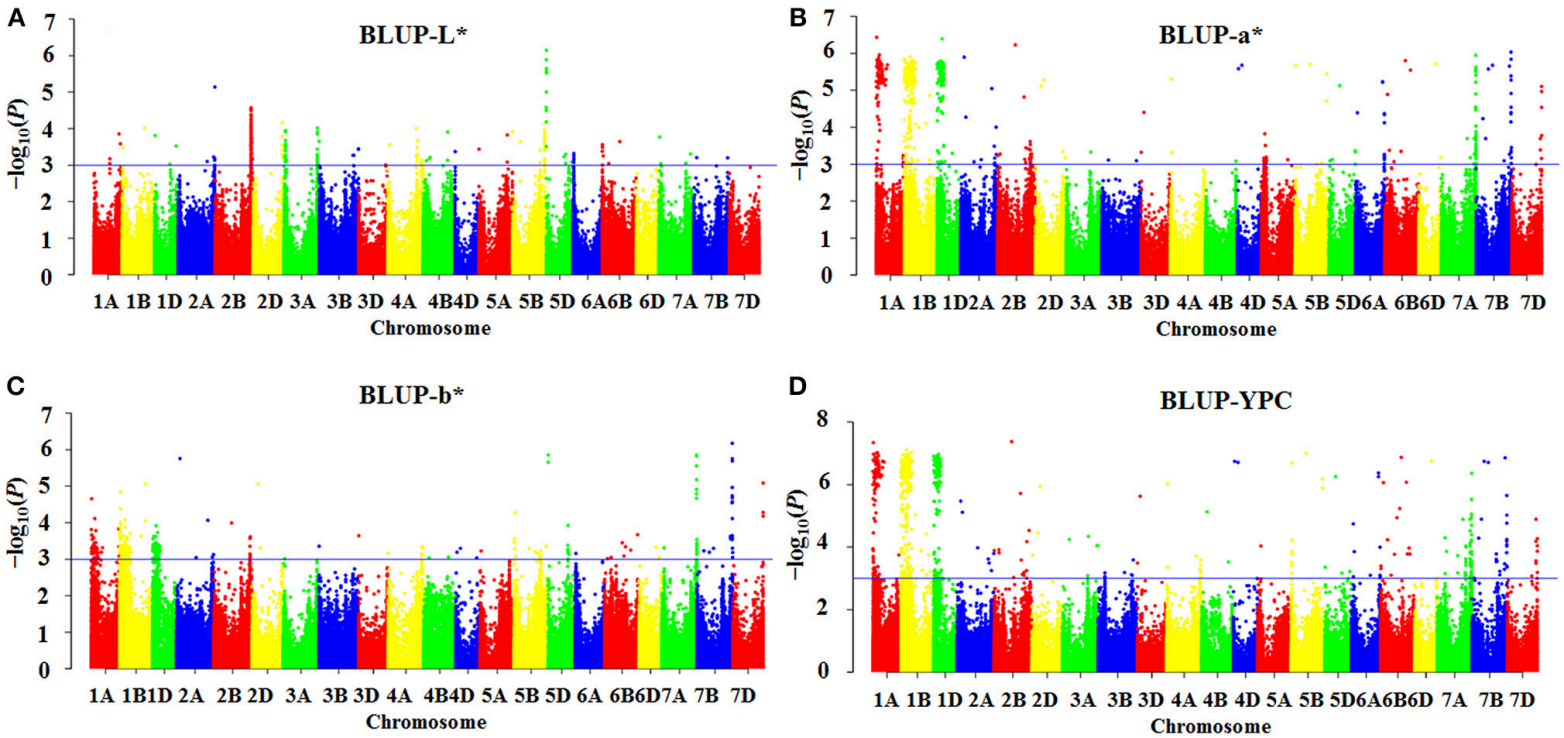
Manhattan plots indicating genomic regions associated with flour color-related traits based on BLUP values. **(A)** L*, **(B)** a*, **(C)** b*, and **(D)** yellow pigment content (YPC). Negative log_10_-transformed *P*-values from a genome-wide scan are plotted against SNP marker positions on each wheat chromosome. The blue horizontal line designates the significant association threshold (–log_10_ *P* ≥3).

As shown in the Q-Q plots (Figure S2), the observed –log_10_ (*P*)-values were closed to the expected distribution, suggesting that the MLM model (Q + K) was appropriate for association analysis of flour color-related traits in this study. One hundred MTAs for flour color-related traits were detected, and 78 were identified in all four environments (Table 2). The highest number of MTAs was detected for YPC (32), followed by a^*^ (28), b^*^ (25) and L^*^ (15). The associations between markers and flour color-related traits are shown by Manhattan plots in Figure 1 and Figure S3.

**Table 2:** Significant marker–trait associations for flour color-related traits using mixed linear model approach.

**Trait**	**Marker^a^**	**Chr^b^**	**Physical position^c^ (bp)**	**Environments^d^**	***P*-value**	***R^2^*^e^ (%)**	**Candidate gene^f^**	**GenBank No**.	**Physical position^c^ (bp)**	**Distance (Mb)**
L^*^	*AX_111611571*	1A	548,493,456–548,493,526	E1, E2, E3, E4, E5	1.40–9.83E^−04^	7.1–9.6	*FPPS2*	JX235715	544,198,065–544,198,296	4.19
	*AX_94634405*	1B	483,114,668–483,114,738	E1, E2, E3, E4, E5	2.95E^−05^-3.71E^−04^	10.1–11.8				
	*AX_94562220*	1D	18,073,435–18,073,505	E1, E3, E4, E5	1.55–7.53E^−04^	7.4–9.5				
	*AX_110044577*	2A	778,128,072–778,128,142	E1, E2, E3, E4, E5	7.29E^−06^-8.74E^−04^	8.1–14.0	*MK*	AFV51837	777,287,545–777,287,970	0.82
	*AX_111612184*	2B	745,494,701–745,494,771	E2, E3, E4, E5	7.52E^−06^-3.30E^−04^	11.0–16.4				
	*IWB26070*	3A	710,831,963–710,832,063	E1, E2, E3, E4, E5	1.25–7.84E^−04^	7.6–10.9				
	*AX_109848219*	4A	29,058,615–29,058,685	E1, E2, E3, E5	2.03–8.72E^−04^	7.5–11.5				
	*AX_111533733*	4A	607,266,755–607,266,825	E1, E2, E4, E5	9.93E-^05^-3.69E^−04^	10.3–12.2				
	*AX_111138030*	4B	524,670,446–524,670,516	E1, E2, E4, E5	1.24–9.80E^−04^	7.2–9.7				
	*AX_111736921*	5A	580,948,231–580,948,301	E1, E3, E4, E5	1.39–3.04E^−04^	9.5–10.7	*LOX2*	GU167921	575,706,041–575,708,873	5.12
	*IWB71821*	5B	669,675,280–669,675,380	E1, E2, E3, E4, E5	1.12–9.81E^−04^	7.9–12.1				
	*Pinb-D1*	5D	3,031,551–303,2419	E1, E2, E3, E4, E5	2.97E^−06^-8.29E^−05^	12.7–17.5				
	*AX_108930866*	5D	417,084,071–417,084,141	E1, E2, E4	3.60–8.47E^−04^	8.6–10.4				
	*AX_109342544*	7A	19,556,781–19,556,851	E1, E2, E3, E4, E5	1.70–9.28E^−04^	7.3–11.5				
	*IWB56095*	7A	644,611,038–644,611,138	E1, E2, E3, E4, E5	2.90–8.01E^−04^	7.3–10.8	*POD*	EU725470	646509653–646510291	1.85
a^*^	*AX_108727598*	1A	7,653,153–7,653,223	E1, E2, E3, E4, E5	7.74E^−08^-2.96E^−06^	14.6–20.0				
	*AX_111611571*	1A	548,493,456–548,493,526	E1, E2, E3, E5	3.01–5.84E^−04^	7.1–7.9	*FPPS2*	JX235715	544,198,065–544,198,296	4.19
	*1B•1R*	1B	-	E1, E2, E3, E4, E5	3.82E^−07^-9.32E^−06^	12.5–16.9				
	*AX_95217104*	1B	540,848,004–540,848,074	E1, E2, E3, E4, E5	1.40-E^−06^-2.30E^−04^	8.3–14.7				
	*IWB9456*	1D	97,314,998–97,315,098	E1, E2, E3, E4, E5	3.56E^−06^-1.44E^−05^	12.0–13.8				
	*AX_95151551*	2A	79,303,812–79,303,882	E1, E2, E3, E4, E5	2.65E^−07^-9.14E^−05^	9.5–17.2				
	*AX_109848219*	2A	629,632,627–629,632,697	E1, E2, E3, E4, E5	2.71E^−06^-1.84E^−04^	8.9–14.9				
	*AX_89310598*	2B	418,276,653–418,276,723	E1, E2, E3, E4, E5	2.27E^−07^-1.22E^−05^	12.1–17.0				
	*AX_111612184*	2B	745,494,701–745,494,771	E1, E2, E3, E4, E5	2.04–9.91E^−04^	7.9–9.9				
	*AX_94596570*	2D	131,312,023–131,312,093	E1, E2, E3, E4, E5	8.82E^−07^-1.62E^−04^	10.7–17.9	*IPPS*	EU783965	187,126,553–187,126,809	8.65
	*AX_108794699*	3D	10,644,700–10,644,770	E1, E2, E3, E4, E5	1.40E^−05^-4.98E^−04^	7.5–11.9				
	*AX_109848219*	4A	29,058,615–29,058,685	E1, E2, E3, E4, E5	1.72E^−06^-6.21E^−05^	10.0–14.3				
	*AX_109058420*	4A	707,784,994–707,785,064	E1, E2, E4	6.78–9.72E^−04^	6.5–11.2	*DXR*	EMS62178	709,122,545–709,122,730	1.31
	*AX_108749277*	4B	606,865,366–606,865,436	E1, E2, E4, E5	6.19–9.72E^−04^	6.7–9.0	*BCH2*	JX171673	608624963–608625486	1.72
	*IWB17540*	4D	26,108,065–26,108,266	E1, E2, E3, E4, E5	9.35E^−07^-3.39E^−05^	10.6–15.0				
	*AX_110042198*	5A	74,535,559–74,535,629	E1, E2, E3, E4, E5	6.37E^−05^-7.32E^−04^	8.9–12.2				
	*AX_110577474*	5B	379,722,302–379,722,372	E1, E2, E3, E4, E5	6.21E^−07^-3.11E^−05^	11.2–16.2				
	*IWB71821*	5B	669,675,280–669,675,380	E1, E2, E3, E4, E5	3.56E^−06^-1.50E^−05^	11.8–13.7				
	*AX_108968610*	5D	321,600,750–321,600,820	E1, E2, E3, E4, E5	2.02E^−06^-1.34E^−04^	9.1–14.5				
	*AX_94533255*	6A	42,879,926–42,879,996	E1, E2, E3, E4, E5	4.07E^−05^-1.59E^−04^	8.7–10.3				
	*AX_111451223*	6A	594,420,538–594,420,608	E1, E2, E3, E4, E5	1.77E^−06^-1.14E^−04^	9.3–14.6				
	*AX_110572276*	6B	99,080,197–99,080,267	E1, E2, E3, E4, E5	7.00E^−06^-1.57E^−04^	9.6–14.1				
	*IWB36240*	6B	437,483,918–437,484,033	E1, E2, E3, E4, E5	9.73E^−07^-1.68E^−05^	11.8–15.6				
	*AX_108940832*	6D	463,914,887–463,914,957	E1, E2, E3, E4, E5	1.22E^−06^-1.76E^−05^	11.5–15.0	*NCED4*	KP099105	355857551-355859437	1.69
	*Psy-A1*	7A	729,328,109–729,323,941	E1, E2, E3, E4, E5	4.02E^−07^-1.31E^−04^	8.9–16.2				
	*AX_110044711*	7B	481,839,758–481,839,828	E1, E2, E3, E4, E5	1.33E^−06^-1.99E^−05^	11.3–14.8				
	*Psy-B1*	7B	739,105,007–739,107,002	E1, E2, E3, E4, E5	1.88E^−07^-5.88E^−05^	10.3–18.2				
	*AX_95195654*	7D	635,560,079–635,560,149	E1, E2, E3, E4, E5	9.74E^−06^-2.37E^−04^	10.5–15.2	*PSY1*	EF600063	636721676-636723811	1.13
b^*^	*AX_108727598*	1A	7,653,153–7,653,223	E1, E2, E3, E4, E5	6.70E^−06^-3.64E^−05^	12.1–14.9				
	*AX_111611571*	1A	548,493,456–548,493,526	E1, E2, E3, E4, E5	1.65–8.07E^−04^	6.9–11.2	*FPPS2*	JX235715	544198065-544198296	4.19
	*1B•1R*	1B	-	E1, E2, E3, E4, E5	1.43E^−05^-1.36E^−04^	8.8–11.8				
	*AX_95217104*	1B	540,848,004–540,848,074	E1, E2, E3, E4, E5	4.22E^−06^-9.00E^−05^	9.6–14.6				
	*AX_94562220*	1D	18,073,435–18,073,505	E1, E2, E3, E4, E5	2.53–8.95E^−04^	9.1–10.5				
	*AX_95151551*	2A	79,303,812–79,303,882	E1, E2, E3, E4, E5	9.60E^−07^-1.47E^−05^	11.9–15.0				
	*AX_109848219*	2A	629,632,627–629,632,697	E1, E2, E3, E5	2.04E^−06^-7.21E^−04^	7.7–15.3				
	*AX_89310598*	2B	418,276,653–418,276,723	E2, E3, E4, E5	1.02–9.19E^−04^	6.7–9.6				
	*AX_111612184*	2B	745,494,701–745,494,771	E2, E3, E4, E5	2.13–5.83E^−04^	9.2–10.5				
	*AX_94596570*	2D	131,312,023–131,312,093	E1, E2, E3, E4, E5	4.62E^−06^-1.14E^−04^	11.5–15.4	*IPPS*	EU783965	187126553-187126809	8.65
	*IWB9681*	3A	37,825,997–37,826,097	E1, E2, E3, E5	5.30–9.97E^−04^	6.6–7.3				
	*IWB38921*	3B	747,712,577–747,712,677	E1, E2, E3	7.07–8.79E^−04^	7.3–7.8				
	*AX_109058420*	4A	707,784,994–707,785,064	E1, E2, E3, E4, E5	2.45–7.05E^−04^	8.9–10.4	*DXR*	EMS62178	709122545-709122730	1.31
	*AX_110952518*	5B	35,290,739–35,290,809	E1, E2, E3, E4, E5	5.92E^−06^-5.58E^−04^	9.5–13.8	*ZISO*	CV770956	36761350-36762789	1.44
	*AX_94508455*	5B	531,581,931–531,582,001	E2, E3, E4, E5	5.17–7.64E^−04^	7.1–7.8				
	*Pinb-D1*	5D	3,031,551–3,032,419	E1, E2, E3, E4, E5	3.36E^−07^-7.21E^−05^	11.7–19.3				
	*AX_108930866*	5D	417,084,071–417,084,141	E2, E3, E4, E5	5.62E^−05^-3.49E^−04^	9.9–12.4				
	*AX_111098507*	6A	17,899,493–17,899,563	E1, E2, E3, E4, E5	2.49–7.15E^−04^	7.5–9.5				
	*AX_109996966*	6B	240,379,787–240,379,857	E1, E2, E3	5.30–8.47E^−04^	7.2–8.5	*LYCB*	JN622196	239283250-239284710	1.07
	*AX_111547031*	6B	689,671,458–689,671,528	E2, E3, E4, E5	2.14–6.33E^−04^	9.2–10.6				
	*AX_111616453*	7A	449,976,248–449,976,318	E1, E2, E3	2.16–6.29E^−04^	8.4–9.1				
	*Psy-A1*	7A	729,328,109–729,323,941	E1, E2, E3, E4, E5	4.07E^−08^-1.74E^−05^	11.7–19.4				
	*AX_94457966*	7B	11,253,471–11,253,541	E1, E2, E3, E4	3.10–8.15E^−04^	7.5–9.0				
	*Psy-B1*	7B	739,105,007–739,107,002	E1, E2, E3, E4, E5	6.47E^−07^-4.34E^−06^	13.5–16.1				
	*AX_95195654*	7D	635,560,079–635,560,149	E1, E2, E3, E4, E5	1.27E^−07^-1.97E^−04^	11.1–20.7	*PSY1*	EF600063	636721676-636723811	1.13
YPC	*AX_108727598*	1A	7,653,153–7,653,223	E1, E2, E3, E4, E5	2.50E^−08^-1.42E^−06^	16.3–20.9				
	*AX_111611571*	1A	548,493,456–548,493,526	E1, E3, E4, E5	1.19E^−05^-7.24E^−04^	8.7–14.1	*FPPS2*	JX235715	544198065-544198296	4.19
	*1B•1R*	1B	-	E1, E2, E3, E4, E5	3.81E^−08^-3.27E^−06^	15.5–20.5				
	*AX_95217104*	1B	540,848,004–540,848,074	E1, E2, E3, E4, E5	1.39E^−05^-2.93E^−04^	7.9–11.6				
	*IWB9456*	1D	97,314,998–97,315,098	E1, E2, E3, E4, E5	2.75E^−08^-3.10E^−06^	15.4–20.7				
	*AX_95151551*	2A	79,303,812–79,303,882	E1, E2, E3, E4	8.66E^−07^-2.36E^−05^	11.1–15.2				
	*AX_110044577*	2A	778,128,072–778,128,142	E1, E2, E3, E4, E5	1.15E^−05^-9.19E^−04^	8.2–14.5	*MK*	AFV51837	777287545-777287970	0.82
	*AX_89310598*	2B	418,276,653–418,276,723	E1, E2, E3, E4, E5	8.34E^−09^-1.14E^−06^	15.0–20.8				
	*AX_111612184*	2B	745,494,701–745,494,771	E1, E2, E3, E4, E5	1.08E^−05^-1.90E^−04^	11.0–15.0				
	*AX_94596570*	2D	131,312,023–131,312,093	E1, E2, E3, E4, E5	2.29E^−07^-1.55E^−05^	11.6–16.3	*IPPS*	EU783965	187126553-187126809	8.65
	*AX_109399477*	3A	549,330,506–549,330,576	E1, E2, E3, E4, E5	1.99E^−05^-2.63E^−04^	9.9–13.9				
	*AX_108968661*	3B	146,971,117–146971187	E1, E3, E4, E5	3.25–9.15E^−04^	6.9–9.9				
	*IWB38921*	3B	747,712,577–747,712,677	E1, E2, E3, E4, E5	1.79–6.92E^−04^	7.7–9.1				
	*AX_108794699*	3D	10,644,700–10,644,770	E1, E2, E3, E4, E5	5.35E^−07^-6.92E^−05^	9.7–15.2				
	*AX_109848219*	4A	29,058,615–29,058,685	E1, E2, E3, E4, E5	2.53E^−07^-2.51E^−05^	11.0–16.2				
	*AX_109058420*	4A	707,784,994–707,785,064	E1, E2, E3, E4, E5	1.06–6.79E^−04^	10.9–13.7	*DXR*	EMS62178	709122545-709122730	1.31
	*AX_108749277*	4B	606,865,366–606,865,436	E1, E2, E3, E4, E5	1.33–8.21E^−04^	8.6–10.4	*BCH2*	JX171673	608624963-608625486	1.72
	*IWB17540*	4D	26,108,065–26,108,266	E1, E2, E3, E4, E5	3.92E^−08^-6.03E^−^06	12.6–18.4				
	*AX_110042198*	5A	74,535,559–74,535,629	E1, E2, E3, E4, E5	6.88E^−05^-2.00E^−04^	10.9–12.0				
	*AX_110577474*	5B	379,722,302–379,722,372	E1, E2, E3, E4, E5	2.82E^−08^-2.69E^−06^	15.4–20.5				
	*IWB71821*	5B	669,675,280–669,675,380	E1, E2, E3, E4, E5	1.57E^−07^-8.57E^−06^	12.8–17.2				
	*AX_108968610*	5D	321,600,750–321,600,820	E1, E2, E3, E4, E5	1.10E^−07^-2.04E^−05^	11.3–17.4				
	*AX_94533255*	6A	42,879,926–42,879,996	E1, E2, E3, E4, E5	4.70E^−06^-1.24E^−04^	8.9–12.4				
	*AX_111451223*	6A	594,420,538–594,420,608	E1, E2, E3, E4, E5	8.85E^−08^-1.69E^−05^	11.5–17.6				
	*AX_110572276*	6B	99,080,197–99,080,267	E1, E2, E3, E4, E5	1.99E^−07^-2.52E^−05^	11.9–18.1				
	*IWB36240*	6B	437,483,918–437,484,033	E1, E2, E3, E4, E5	3.18E^−08^-3.43E^−06^	14.3–19.8				
	*AX_108940832*	6D	463,914,887–463,914,957	E1, E2, E3, E4, E5	3.65E^−08^-4.87E^−06^	13.1–18.8	*NCED4*	KP099105	355857551-355859437	1.69
	*AX_111136917*	7A	548,316,594–548,316,664	E1, E2, E3, E4, E5	5.33E^−06^-6.75E^−05^	9.2–12.9				
	*Psy-A1*	7A	729,328,109–729,323,941	E1, E2, E3, E4, E5	9.43E^−07^-1.98E^−05^	11.2–15.0				
	*AX_110044711*	7B	481,839,758–481,839,828	E1, E2, E3, E4, E5	4.11E^−08^-5.55E^−06^	12.7–18.5				
	*Psy-B1*	7B	739,105,007–739,107,002	E1, E2, E3, E4, E5	9,89E^−07^-1.43E^−05^	12.0–15.7				
	*AX_95195654*	7D	635,560,079–635,560,149	E1, E2, E3, E4, E5	3.12E^−05^-2.12E^−04^	10.8–13.5	*PSY1*	EF600063	636721676-636723811	1.13

a *Representative marker at the MTA*.

b *Chromosome*.

c *The physical positions of SNP markers based on wheat genome sequences from the International Wheat Genome Sequencing Consortium (IWGSC RefSeq v1.0, http://www.wheatgenome.org/)*.

d *E1, 2012-2013 AY; E2, 2012-2013 SX; E3, 2013-2014 AY; E4, 2013-2014 SX; E5, BLUP, a best linear unbiased predictor of flour color-related traits in 166 common wheat cultivars across four environments*.

e *The percentage of variation explained by each locus*.

f *FPPS2, Farnesyl pyrophosphate synthase 2; MK, mevalonate kinase; LOX2, lipoxygenase 2; POD, peroxidase; IPPS, Isopentenyl pyrophosphate isomerase; DXR, 1-deoxy-D-xylulose 5-phosphate reductoisomerase; BCH2, Carotenoid β-ring hydroxylase 2; NCED4, 9-cis-epoxycarotenoid dioxygenase 4; PSY1, Phytoene synthase 1; ZISO, Cis-zeta-carotene isomerase; LCYB, Lycopene β-cyclase*.

MTAs of brightness were detected on chromosomes 1A, 1B, 1D, 2A, 2B, 3A, 4A (2), 4B, 5A, 5B, 5D (2), and 7A (2), each explaining 7.1–17.5% of the phenotypic variation across environments (Table 2). The MTAs on chromosomes 1D, 2B, 4A (2), 4B, 5A and 5D (*AX_108930866*) were identified in three environments, and the remaining MTAs were detected in all four environments. The two most significant markers were *AX_111612184* on chromosome 2B and *Pinb-D1* on chromosome 5D, explaining 11.0–16.4% and 12.7–17.5% of phenotypic variation, respectively.

For redness, the MTAs were distributed on chromosomes 1A (2), 1B (2), 1D, 2A (2), 2B (2), 2D, 3D, 4A (2), 4B, 4D, 5A, 5B (2), 5D, 6A (2), 6B (2), 6D, 7A, 7B (2) and 7D, individually explaining 6.5–20.0% of the phenotypic variation (Table 2). The loci on chromosomes 1A (*AX_111611571*), 4A (*AX_109058420*) and 4B were identified in three environments, and the other 25 MTAs were significantly associated with a^*^ across all four environments. The MTA on chromosome 1A (*AX_108727598*) showed the strongest association with a^*^, explaining phenotypic variation ranging from 14.6 to 20.0%.

Yellowness MTAs, located on chromosomes 1A (2), 1B (2), 1D, 2A (2), 2B (2), 2D, 3A, 3B, 4A, 5B (2), 5D (2), 6A, 6B (2), 7A (2), 7B (2) and 7D, explained phenotypic variation ranging from 6.6 to 20.7% (Table 2). The loci on chromosomes 2A (*AX_109848219*), 2B (2), 3A, 3B, 5B (*AX_94508455*), 5D (*AX_108930866*), 6B (2) and 7A (*AX_111616453*) were detected in three environments, whereas the others were significant for b^*^ in all four environments. The MTAs on chromosomes 2A (2), 2D and 7D, and *Pinb-D1, Psy-A1* and *Psy-B1* showed stronger association with b^*^, explaining more than 15% phenotypic variation in some environments.

For yellow pigment content, we detected MTAs on all chromosomes, explaining 6.9–20.9% of the total variation (Table 2). MTAs on chromosomes 1A (*AX_111611571*) and 3B (*AX_108968661*) were identified in three environments, while the remaining were observed in all four environments. The MTAs on chromosomes 1A (*AX_108727598*), 1D, 2B (*AX_89310598*), 5B (*AX_110577474*) and 1B•1R translocation showed stronger association with YPC than other MTAs.

### *In silico* prediction of candidate genes

The putative biological functions of 1,543 significant SNP markers associated with flour color-related traits identified in the present study were assigned (data not shown). Briefly, *AX_109030196*, associated with a^*^, b^*^ and YPC, corresponds to premnaspirodiene oxygenase (*PO*) gene involved in sesquiterpenoid and triterpenoid biosynthesis (Table 3). For the MTA on chromosome 1D (97,314,998–97,315,098 Mb), two candidate genes for a^*^ and YPC were identified; one is a putative gene (*IWB35120*) encoding the dihydrolipoyl dehydrogenase (DLD) enzyme, and the other (*IWB31766*) corresponds to leghemoglobin reductase (LBR), both involved in glycolysis/gluconeogenesis. Either one or even both genes are potential candidates for the observed MTA in this genomic region. *AX_111471334*, corresponding to phosphomannomutase (*PMM*) gene, is a potential candidate gene for the MTA on chromosome 3B (146,971,117–146,971,187 Mb) for YPC. *IWB36240*, significant for a^*^ and YPC, corresponds to 4-hydroxy-3-methylbut-2-en-1-yl diphosphate synthase (*MDPS*) gene involved in terpenoid backbone biosynthesis.

**Table 3:** Candidate genes identified by putative biological functions of significant SNP markers.

**MTAs**				**Candidate genes**				**Distance (Mb)**
**Trait**	**Marker^a^**	**Chr^b^**	**Physical position^c^ (bp)**	**Marker^d^**	**Physical position^c^ (bp)**	**Candidate gene (bp)^e^**	**Pathway**	
a^*^, b^*^, YPC	*AX_108727598*	1A	7,653,153–7,653,223	*AX_109030196*	10,126,611–10,126,681	*PO*	Sesquiterpenoid and triterpenoid biosynthesis	2.42
a^*^, YPC	*IWB9456*	1D	97,314,998–97,315,098	*IWB35120*	100,325,418–100,325,536	*DLD*	Glycolysis/Gluconeogenesis	2.94
a^*^, YPC	*IWB9456*	1D	97,314,998–97,315,098	*IWB31766*	103,350,946–103,351,043	*LBR*	Glycolysis/Gluconeogenesis	5.89
YPC	*AX_108968661*	3B	146,971,117–146,971,187	*AX_111471334*	146,551,873–146,551,943	*PMM*	Glycolysis/Gluconeogenesis	0.41
a^*^, YPC	*IWB36240*	6B	437,483,918–437,484,033	*IWB36240*	437,483,918–437,484,033	*MDPS*	Terpenoid backbone biosynthesis	0.00

a *Representative marker at the MTA*.

b *Chromosome*.

c *The physical positions of SNP markers based on wheat genome sequences from the International Wheat Genome Sequencing Consortium (IWGSC RefSeq v1.0, http://www.wheatgenome.org/)*.

d *significant SNP marker*.

e *PO, Premnaspirodiene oxygenase; DLD, Dihydrolipoyl dehydrogenase; LBR, Leghemoglobin reductase; PMM, Phosphomannomutase; MDPS, 4-hydroxy-3-methylbut-2-en-1-yl diphosphate synthase*.

Based on the reference wheat genome sequences from the IWGSC RefSeq v1.0, the 40 known genes influencing flour color-related traits were mapped on the physical map (Table S5). Briefly, the farnesyl pyrophosphate synthase 2 gene (*FPPS2*) was located about 4.19 Mb from *AX_111611571* significant for L^*^, a^*^, b^*^ and YPC (Table 2). *AX_94596570* (2D), *AX_109058420* (4A) and *AX_95195654* (7D) associated with a^*^, b^*^ and YPC was located about 8.65, 1.31 and 1.13 Mb from isopentenyl pyrophosphate isomerase (*IPPS*), 1-deoxy-D-xylulose 5-phosphate reductoisomerase (*DXR*) and *PSY1* genes, respectively. The mevalonate kinase (*MK*) gene on chromosome 2A was located in close physical proximity with *AX_110044577* (0.82 Mb) associate with both L^*^ and YPC. *AX_108749277* (4B) and *AX_108940832* (6D) significant for a^*^ and YPC was located 1.72 and 1.69 Mb from carotenoid β-ring hydroxylase 2 (*BCH2*) and 9-cis-epoxycarotenoid dioxygenase 4 (*NCED4*) genes, respectively. The lipoxygenase 2 gene (*LOX2*) on chromosome 5A was 5.12 Mb from *AX_111736921* controlling L^*^. *AX_111736921* significant for L^*^ on chromosome 7A was close to peroxidase gene (*POD*) (1.85 Mb). The *cis*-zeta-carotene isomerase gene (*ZISO*) on chromosome 5B was detected at a distance of 1.44 Mb from *AX_110952518* associated with b^*^. *AX_109996966* significant for b^*^ was located about 1.07 Mb proximal to the lycopene β-cyclase gene (*LYCB*). Notably, most of candidate genes, except for *LOX2, POD, ZISO, LCYB* and *PMM*, controlled more than one flour color-related traits.

## Discussion

### Detection of QTL by GWAS

GWAS has become an efficient tool for genetic dissection of complex traits. Among the diverse accessions analyzed in this study, substantial phenotypic variation in flour color-related traits indicated a wide range of genetic diversity (Table 1, Figure S1). Based on diverse genotypes and high-throughput SNP arrays, GWAS was a powerful strategy for dissecting flour color-related traits and identifying candidate genes.

Spurious associations are often the result of structured relationships within the population and may be reduced by taking population structure into account (Pritchard et al., 2000; Yu et al., 2006). Therefore, assessment of population structure is important prior to conducting a GWAS. As described in Liu et al. (2017) the core collection in this study was structured into three subpopulations, largely in agreement with their geographical origin. This pattern can be caused by several factors, including disproportional usage of a limited number of founders in developing regional populations and enrichment of alleles associated with regional adaptation by local breeding programs.

As shown in Q-Q plots (Figure S2), MLM greatly reduced spurious associations. Consistency across environments was used as an additional criterion for MTAs significant at FDR < 0.05 to reduce the risk of false marker–trait associations. Finally, the 100 MTAs for flour color-related traits were identified in at least three environments, which will be useful for metabolomic studies of flour color-related traits. The more robust markers can then be implemented in breeding programs to ensure that increasingly stringent color requirements imposed by industry are met through early screening of breeding lines. Importantly, the effects of these MTAs and consequent values for selection in breeding programs require validation in bi-parental populations.

### Correlations among flour color-related traits

Pearson correlation coefficients among all flour color-related traits showed that b^*^ was negatively correlated with brightness (*r* = −0.71) and redness (*r* = −0.68) and yellow pigment content was negatively correlated with redness (*r* = −0.89) and positively correlated with yellowness (*r* = 0.83). As expected the strong phenotypic correlations among flour color-related traits were generally based on a large number of shared MTAs (Table 2). b^*^ had five and 13 MTAs in common with L^*^ and a^*^, respectively. YPC shared 27 and 13 same genetic regions with a^*^ and b^*^, respectively. Of course, some loci were also identified specific for L^*^, a^*^, b^*^ and YPC, respectively, indicating that flour color-related traits could be controlled by common major-effect loci, but also modified by numerous trait-specific loci.

To improve flour color-related traits, using multi-trait markers in MAS may increase QTL pyramiding efficiency. Most Chinese foods, such as steamed buns and noodles, typically require a white or creamy flour type (higher L^*^) with lower b^*^ and YPC. In this situation, these multi-trait regions could be important targets to reduce b^*^ and YPC and increase L^*^ simultaneously.

### Comparison with previous studies

As shown in Table 2, known major genes controlling flour color-related traits and previously identified in bi-parental mapping populations were confirmed in the present study, such as *Psy-A1, Psy-B1, Pinb-D1* and the 1B•1R translocation (Nagamine et al., 2003; He et al., 2008, 2009; Tsilo et al., 2011). On average, cultivars carrying the *Psy-A1b* allele produced significantly lower b^*^ (7.48 ± 1.53) and YPC (0.87 ± 0.30 μg.g^−1^) than lines carrying the *Psy-A1a* allele (9.56 ± 1.84 and 1.34 ± 0.39 μg.g^−1^), while higher a^*^ was associated with the *Psy-A1b* allele (−0.66 ± 0.28 vs. −0.97 ± 0.32). b^*^ (11.52 ± 1.55) and YPC (1.77 ± 0.34 μg.g^−1^) values for 19 cultivars with the *Psy-B1a* allele were significantly higher than those of 146 lines with *Psy-B1b* (8.46 ± 1.75 and 1.09 ± 0.36 μg.g^−1^), whereas lines carrying the *Psy-B1b* allele generated higher a^*^ (−0.80 ± 0.30 vs. −1.32 ± 0.27). For *Pinb-D1*, lines with the *Pinb-D1b* allele produced significantly lower L^*^ (89.95 ± 0.56) and higher b^*^ (9.64 ± 1.76) than those with *Pinb-D1a* (90.75 ± 1.12 and 7.67 ± 1.70). Cultivars with the 1B•1R translocation had significantly higher b^*^ (9.65 ± 1.03) and YPC (1.39 ± 0.44 μg.g^−1^) than those without (8.05 ± 0.91) and (0.97 ± 0.29 μg.g^−1^), while non-1B•1R translocation genotypes were associated with higher a^*^ (−1.02 ± 0.34 vs. −0.72 ± 0.28) (data not shown). These results confirmed that *Psy-A1, Psy-B1, Pinb-D1* and the 1B•1R translocation were major-effect loci for flour color-related traits, and can be considered as stable and useful for MAS in breeding programs. However, *e-Lcy3A, Talcye-B1, TaPds-B1* and *Lox-B1* were not significantly associated with flour color-related traits as they have been reported previously (Dong, 2011; Geng et al., 2012; Crawford and Francki, 2013b). This might be due to the genetic background of the germplasm panel used in this study. More accessions with wider genetic variation will be collected and analyzed in further research.

Because no QTL for L^*^ was previously mapped to chromosome 7A (2), the two MTAs in this work may be new loci associated with this trait. Similarly, MTA for a^*^ located on chromosome 2A (629,632,627–629,632,697 Mb) and MTA for YPC on chromosome 7D (635,560,079–635,560,149 Mb) were not matched to any previously identified QTL. Hence these loci are likely to be new and should be attractive candidate regions on which to focus further in dissecting the genetic architecture of flour color-related traits in wheat. Notably, MTAs on chromosomes 1A (7,653,153–7,653,223 Mb) and 2B (418,276,653–418,276,723 Mb) showed stronger association with a^*^ than *Psy-A1* and *Psy-B1* in at least three environments (data not shown). Similarly, 11 MTAs on chromosomes 1A (7,653,153–7,653,223 Mb), 1D, 2B (418,276,653–418,276,723 Mb), 4D, 5B (379722302–379722372 Mb), 5D, 6A (594,420,538–594,420,608 Mb), 6B (2), 6D, and 7B (481,839,758–481,839,828 Mb) explained much more phenotypic variation of YPC than *Psy-A1* and *Psy-B1* in at least three environments (data not shown). These demonstrated that GWAS is a powerful mapping approach for identifying genomic regions underlying variation in flour color based on diverse genotypes and high-throughput SNP arrays; they also confirmed previously detected QTL and revealed novel QTL that were not found in bi-parental populations previously.

### Candidate genes

Knowledge of the functions of significant SNP markers provides a very useful tool for identification of candidate genes for traits under investigation. Based on a comparative genomics approach, annotations of flanking sequences of significant SNP markers predicted that some genomic regions encode proteins that are important components of pathways linked to carotenoid biosynthesis and degradation (Table 3). For example, PO (*AX_109030196*) participated in sesquiterpenoid and triterpenoid biosynthesis; DLD (*IWB35120*), LBR (*IWB31766*) and PMM (*AX_111471334*) were involved in glycolysis/gluconeogenesis; and MDPS (*IWB36240*) played a role in terpenoid backbone biosynthesis. These genes can be considered potential candidate genes for flour color-related traits.

Over the past decades, the gene discovery in bread wheat has been largely limited due to the absence of reference genome sequences. However, recent advances in high-throughput genotyping platforms and publicly available wheat genome sequences offer researchers new opportunities to achieve their goals. Based on the reference wheat genome sequences from the IWGSC RefSeq v1.0, 40 known genes influencing flour color-related traits were assigned to chromosome locations (Table S5). Eleven of the 40 known genes were located close to 23 MTAs for flour color-related traits (Table 2), including four terpenoid backbone biosynthesis genes (*FPPS2, IPPS, DXR* and *MK*), five genes involved in carotenoid biosynthesis (*ZISO, LYCB, PSY-D1, BCH2* and *NCED4*), and two related to carotenoid degradation (*LOX2* and *POD*). Therefore, the co-linearity of 11 known genes and 23 MTAs suggested that these might be candidate genes for flour color-related traits. In fact, all these candidate genes should be confirmed in bi-parental population mapping or by reverse genetic approaches.

Out of 40 known genes blasted against the available dataset of SNP marker sequences (Wang et al., 2014), SNP markers corresponded to nine genes, including *FPPS1, POD, FPPS2, MK*, aldehyde oxidase 3, carotenoid β-ring hydroxylase, geranylgeranyl transferase I β-subunit, geranylgeranyl transferase I α-subunit and catalase 3 (Table S6). However, only *FPPS2, POD* and *MK* were significantly associated with the flour color-related traits, based on chromosome locations and physical distances. Briefly, cultivars carrying the “A” SNP in *FPPS2* produced significantly lower L^*^ (90.20 ± 0.90), a^*^ (−0.91 ± 0.34) and higher b^*^ (9.17 ± 1.95) and YPC (1.25 ± 0.42) than those with the “G” (90.44 ± 0.93, −0.78 ± 0.33, 8.24 ± 1.88 and 1.05 ± 0.38). Seventy-four cultivars with the “A” SNP in *POD* showed significantly lower L^*^ (90.05 ± 0.90) than those with “G” (90.48 ± 0.88). For *MK*, lines with the “C” SNP had significantly higher L^*^ (90.75 ± 1.02) and lower YPC (1.09 ± 0.26) than those with the “G” (90.25 ± 0.90 and 1.18 ± 0.43).

Based on gene ontology analysis, all these 16 candidate genes are related to four main biological processes. Briefly, *PMM* is involved in GDP-mannose biosynthetic process; *MDPS, FPPS2, IPPS, DXR* and *MK* participate in the isoprenoid/terpenoid biosynthetic process; *ZISO, LYCB, PSY* and *BCH2* involve in the carotenoid biosynthetic/carotene process; and *PO, DLD, LBR, NCED4, LOX2* and *POD* are related to the oxidation-reduction process; these are corresponding to carotenoid precursor supply, carotenoid biosynthesis and carotenoid degradation, respectively (Rodríguez-Concepción et al., 2001; Qin et al., 2012; Zeng et al., 2015).

### Effects of favorable alleles on flour color-related traits

**Figure 2:**
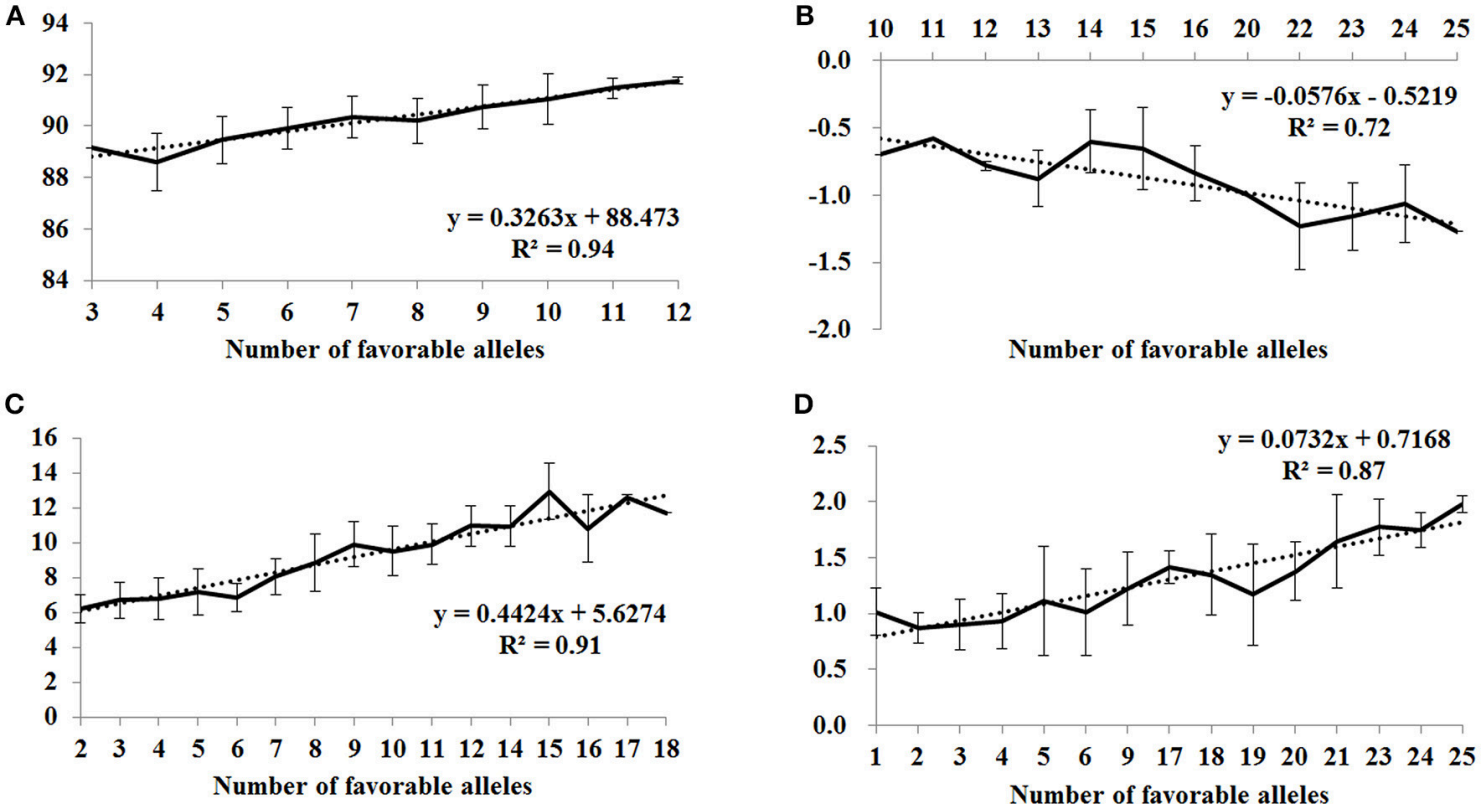
Linear regressions between number of favorable alleles and BLUP values of flour color-related traits. **(A)** L*, **(B)** a*, **(C)** b*, and **(D)** yellow pigment content (YPC).

To assess the pyramiding effect of favorable alleles of MTAs for flour color-related traits, we examined the number of favorable alleles in each accession, and the BLUP value for each trait was regressed against the number of favorable alleles. The favorable alleles of significantly associated SNPs showed additive effects on brightness, redness, yellowness and yellow pigment content (Figure 2). As the number of favorable alleles increased, L^*^, b^*^ and YPC values also increased and a^*^ decreased, and linear regressions (R^2^) between numbers of favorable alleles and phenotype were 0.94, 0.72, 0.91 and 0.87, respectively. Higher b^*^ and YPC are desirable for yellow alkaline noodles and human health, while most Chinese foods typically require a white or creamy flour type with lower b^*^ and YPC. In this situation, cultivars with minimum (Wennong5, Lumai21, Jinan17 and Funo) or maximum (Huaimai18, Zhongmai875, Shan715, Lumai11 and Jinmai61) numbers of favorable alleles for b^*^ and YPC can be used as breeding parents to achieve defined color and nutritional properties of end-use products.

## Conclusions

We performed a genome-wide association analysis on 166 bread wheat cultivars using the wheat 90 and 660 K SNP arrays and 10 allele-specific markers, and identified 100 MTAs for flour color-related traits. Broad comparison of MTAs identified in this study with QTL in previous reports indicated many common loci conditioning flour color-related traits, and four MTAs detected were new, including MTAs on chromosome 7A (2) for L^*^, chromosome 2A (629,632,627–629,632,697 Mb) for a^*^ and chromosome 7D (635,560,079–635,560,149 Mb) for YPC. Two and 11 loci explained much more phenotypic variation of a^*^ and YPC than phytoene synthase 1 gene (*Psy1*), respectively. Based on biochemical information and bioinformatics analyses 16 predicted candidate genes were related to carotenoid biosynthesis and degradation, terpenoid backbone biosynthesis and glycolysis/gluconeogenesis. We will confirm these candidate genes in bi-parental populations and do some gene function analysis in the future.

The genomic regions associated with flour color-related traits identified in this study bring new insights to understanding the genetic basis of these traits, and new markers are useful for wheat quality improvement by MAS. Moreover, the candidate genes may serve as promising targets for study of the molecular mechanisms underlying flour color-related traits in wheat. This study also confirmed that GWAS is a powerful approach to validate known genes for complex traits and identify novel loci.

## Author contributions

SZ performed the experiment and wrote the paper. SZ, JL, and DX analyzed data. WW, JY, PZ, and YW participated in the field trials. YH, WM, and SC assisted in writing the paper. ZH and XX designed the experiment and wrote the paper. All authors read and approved the final manuscript.

### Conflict of interest statement

The authors declare that the research was conducted in the absence of any commercial or financial relationships that could be construed as a potential conflict of interest.

## Abbreviations

a^*^: Redness
b^*^: Yellowness
*BCH2*: Carotenoid β-ring hydroxylase 2
BLUP: Best linear unbiased predictor
*DLD*: Dihydrolipoyl dehydrogenase
*DXR*: 1-deoxy-D-xylulose 5-phosphate reductoisomerase
*FPPS2*: Farnesyl pyrophosphate synthase 2
GWAS: Genome-wide association study
*h*^2^: Broad-sense heritability
*IPPS*: Isopentenyl pyrophosphate isomerase
IWGSC: International Wheat Genome Sequencing Consortium
K: Genetic relatedness
L^*^: Brightness
*LBR*: Leghemoglobin reductase
*LCYB*: Lycopene β-cyclase
LD: Linkage disequilibrium
LOESS: Locally weighed polynomial regression
*LOX2*: Lipoxygenase 2
MAF: Minor allele frequency
MAS: Marker-assisted selection
*MDPS*: 4-hydroxy-3-methylbut-2-en-1-yl diphosphate synthase
MLM: Mixed linear model
*MK*: Mevalonate kinase
MTAs: Marker–trait associations
*NCED4*: 9-cis-epoxycarotenoid dioxygenase 4
*PMM*: Phosphomannomutase
*PO*: Premnaspirodiene oxygenase
*POD*: Peroxidase
*Psy1*: Phytoene synthase 1
Q: Population structure
Q-Q: quantile-quantile
QTL: Quantitative trait loci
*r*: Correlation coefficient
*R*^2^: Percentage of variation explained by each locus
YPC: Yellow pigment content
*ZISO*: *Cis*-zeta-carotene isomerase.
