# Supplementary material for: A Genome-Wide Association Study Reveals a Rich Genetic Architecture of Flour Color-Related Traits in Bread Wheat
Source: Front Plant Sci. 2018 Aug 3;9:1136. doi: 10.3389/fpls.2018.01136 (PMC6085589; doi:10.3389/fpls.2018.01136)
Supplement: Supplementary file 1 [file Table_1.DOCX]

**Table S1** Information of the 166 bread wheat cultivars and their subgroup as defined by STRUCTURE analysis

| **No.** | **Name** | **Origin** | **Subgroup** |
| --- | --- | --- | --- |
| 1 | An 1331 | Anhui | 2 |
| 2 | Fu 936 | Anhui | 2 |
| 3 | Huaimai 18 | Anhui | 1 |
| 4 | Huaimai 20 | Anhui | 1 |
| 5 | Huaimai 21 | Anhui | 3 |
| 6 | Su 0663 | Anhui | 2 |
| 7 | Sunong 6 | Anhui | 2 |
| 8 | Wan 23094 | Anhui | 2 |
| 9 | Wanmai 19 | Anhui | 2 |
| 10 | Wanmai 29 | Anhui | 2 |
| 11 | Wanmai 33 | Anhui | 2 |
| 12 | Wanmai 38 | Anhui | 1 |
| 13 | Wanmai 50 | Anhui | 2 |
| 14 | Wanmai 52 | Anhui | 2 |
| 15 | Wanmai 53 | Anhui | 2 |
| 16 | Gaocheng 8901 | Hebei | 1 |
| 17 | Gaoyou 503 | Hebei | 2 |
| 18 | Han 6172 | Hebei | 3 |
| 19 | Heng 7228 | Hebei | 3 |
| 20 | Hengguan 33 | Hebei | 3 |
| 21 | Hengguan 35 | Hebei | 3 |
| 22 | Jinhe 9123 | Hebei | 3 |
| 23 | Jishi 02-1 | Hebei | 2 |
| 24 | Shi 4185 | Hebei | 3 |
| 25 | Shijiazhuang 15 | Hebei | 3 |
| 26 | Shijiazhuang 8 | Hebei | 3 |
| 27 | Shixin 733 | Hebei | 2 |
| 28 | Shixin 828 | Hebei | 2 |
| 29 | Shiyou 17 | Hebei | 1 |
| 30 | 11CA40 | Henan | 1 |
| 31 | 85Zhong 33 | Henan | 3 |
| 32 | Aikang 58 | Henan | 3 |
| 33 | Bainong 3217 | Henan | 2 |
| 34 | Bainong 64 | Henan | 2 |
| 35 | Huapei 5 | Henan | 3 |
| 36 | Lankao 2 | Henan | 3 |
| 37 | Lankao 24 | Henan | 3 |
| 38 | Lankao 906 | Henan | 3 |
| 39 | Luohan 2 | Henan | 2 |
| 40 | Luomai 21 | Henan | 3 |
| 41 | Neixiang 188 | Henan | 2 |
| 42 | Neixiang 5 | Henan | 3 |
| 43 | St1472/506 | Henan | 2 |
| 44 | Xinmai 19 | Henan | 2 |
| 45 | Xinmai 9 | Henan | 2 |
| 46 | Xinmai 9408 | Henan | 2 |
| 47 | Yanzhan 4110 | Henan | 2 |
| 48 | Yumai 13 | Henan | 1 |
| 49 | Yumai 18 | Henan | 2 |
| 50 | Yumai 2 | Henan | 3 |
| 51 | Yumai 21 | Henan | 1 |
| **No.** | **Name** | **Origin** | **Subgroup** |
| 52 | Yumai 34 | Henan | 1 |
| 53 | Yumai 35 | Henan | 3 |
| 54 | Yumai 47 | Henan | 2 |
| 55 | Yumai 49 | Henan | 2 |
| 56 | Yumai 50 | Henan | 3 |
| 57 | Yumai 57 | Henan | 2 |
| 58 | Yumai 63 | Henan | 2 |
| 59 | Yumai 7 | Henan | 3 |
| 60 | Zheng 9023 | Henan | 2 |
| 61 | Zhengmai 366 | Henan | 2 |
| 62 | Zhengzhou 3 | Henan | 2 |
| 63 | Zhong 892 | Henan | 3 |
| 64 | Zhongmai 871 | Henan | 3 |
| 65 | Zhongmai 875 | Henan | 3 |
| 66 | Zhongmai 895 | Henan | 3 |
| 67 | Zhongyu 5 | Henan | 3 |
| 68 | Zhongyu 9 | Henan | 3 |
| 69 | Zhou8425B | Henan | 3 |
| 70 | Zhoumai 11 | Henan | 3 |
| 71 | Zhoumai 12 | Henan | 3 |
| 72 | Zhoumai 13 | Henan | 3 |
| 73 | Zhoumai 16 | Henan | 3 |
| 74 | Zhoumai 18 | Henan | 3 |
| 75 | Zhoumai 19 | Henan | 2 |
| 76 | Zhoumai 22 | Henan | 3 |
| 77 | Zhoumai 23 | Henan | 2 |
| 78 | Zhoumai 25 | Henan | 3 |
| 79 | Zhoumai 26 | Henan | 3 |
| 80 | Zhoumai 28 | Henan | 3 |
| 81 | Zhoumai 30 | Henan | 3 |
| 82 | Zhoumai 31 | Henan | 2 |
| 83 | Zhoumai 32 | Henan | 3 |
| 84 | Aifeng 3 | Shanxi | 1 |
| 85 | Bima 1 | Shaanxi | 1 |
| 86 | Bima 4 | Shaanxi | 1 |
| 87 | Fengchan 3 | Shaanxi | 2 |
| 88 | Shan 150 | Shaanxi | 2 |
| 89 | Shan 229 | Shaanxi | 2 |
| 90 | Shan 253 | Shaanxi | 2 |
| 91 | Shan 354 | Shaanxi | 3 |
| 92 | Shan 512 | Shaanxi | 2 |
| 93 | Shan 715 | Shaanxi | 3 |
| 94 | Shanmai 509 | Shaanxi | 3 |
| 95 | Shanmai 94 | Shaanxi | 2 |
| 96 | Shannong 7859 | Shaanxi | 3 |
| 97 | Shannong 981 | Shaanxi | 2 |
| 98 | Shanyou 225 | Shaanxi | 2 |
| 99 | Wunong 148 | Shaanxi | 2 |
| 100 | Xiaoyan 22 | Shaanxi | 3 |
| 101 | Xiaoyan 54 | Shaanxi | 2 |
| 102 | Xiaoyan 6 | Shaanxi | 2 |
| 103 | Xiaoyan 81 | Shaanxi | 2 |
| 104 | Xinong 1376 | Shaanxi | 3 |
| 105 | Xinong 2000-7 | Shaanxi | 2 |
| **No.** | **Name** | **Origin** | **Subgroup** |
| 106 | Xinong 291 | Shaanxi | 1 |
| 107 | Xinong 88 | Shaanxi | 2 |
| 108 | Xinong 979-005 | Shaanxi | 2 |
| 109 | Jimai 19 | Shandong | 1 |
| 110 | Jimai 20 | Shandong | 1 |
| 111 | Jimai 21 | Shandong | 1 |
| 112 | Jimai 22 | Shandong | 1 |
| 113 | Jinan 13 | Shandong | 1 |
| 114 | Jinan 17 | Shandong | 1 |
| 115 | Jining 16 | Shandong | 1 |
| 116 | Liangxing 66 | Shandong | 1 |
| 117 | Liangxing 99 | Shandong | 1 |
| 118 | Linmai 2 | Shandong | 1 |
| 119 | Linmai 4 | Shandong | 1 |
| 120 | Luami 15 | Shandong | 3 |
| 121 | Lumai 11 | Shandong | 1 |
| 122 | Lumai 14 | Shandong | 1 |
| 123 | Lumai 21 | Shandong | 1 |
| 124 | Lumai 23 | Shandong | 1 |
| 125 | Lumai 5 | Shandong | 1 |
| 126 | Lumai 6 | Shandong | 2 |
| 127 | Lumai 7 | Shandong | 3 |
| 128 | Lumai 8 | Shandong | 1 |
| 129 | Lumai 9 | Shandong | 1 |
| 130 | Luyuan 502 | Shandong | 1 |
| 131 | PH 82-2 | Shandong | 2 |
| 132 | Shannong 20 | Shandong | 1 |
| 133 | Taishan 1 | Shandong | 1 |
| 134 | Taishan 5 | Shandong | 1 |
| 135 | Wennong 14 | Shandong | 1 |
| 136 | Wennong 5 | Shandong | 1 |
| 137 | Yannong 15 | Shandong | 1 |
| 138 | Yannong 18 | Shandong | 3 |
| 139 | Yannong 19 | Shandong | 1 |
| 140 | Zimai 12 | Shandong | 1 |
| 141 | Zixuan 2 | Shandong | 1 |
| 142 | Jinmai 61 | Shanxi | 1 |
| 143 | Linhan 2 | Shanxi | 2 |
| 144 | Linkang 12 | Shanxi | 3 |
| 145 | Aca 601 | Argentina | 1 |
| 146 | Aca 801 | Argentina | 1 |
| 147 | Klein Flecha | Argentina | 1 |
| 148 | Klein Jabal1 | Argentina | 1 |
| 149 | Nidera Baguette 10 | Argentina | 1 |
| 150 | Nidera Baguette 20 | Argentina | 1 |
| 151 | Prointa Colibr 1 | Argentina | 1 |
| 152 | Sunstate | Australia | 1 |
| 153 | Abbondanza | Italy | 1 |
| 154 | Barra | Italy | 1 |
| 155 | Dorico | Italy | 1 |
| 156 | Funo | Italy | 2 |
| 157 | Genio | Italy | 1 |
| 158 | Lampo | Italy | 1 |
| 159 | Libero | Italy | 1 |
| **No.** | **Name** | **Origin** | **Subgroup** |
| 160 | Mantol | Italy | 1 |
| 161 | Sagittario | Italy | 1 |
| 162 | Kanto 107 | Japan | 1 |
| 163 | Kitanokaori | Japan | 3 |
| 164 | Norin 61 | Japan | 1 |
| 165 | Norin 67 | Japan | 1 |
| 166 | Hk1/6/Nvsr3/5/Bez/Tvr/5/Cfn  /Bez//Su92/Ci13645/3Nai60 | Turkey | 1 |
