# Supplementary material for: A Genome-Wide Association Study Reveals a Rich Genetic Architecture of Flour Color-Related Traits in Bread Wheat
Source: Front Plant Sci. 2018 Aug 3;9:1136. doi: 10.3389/fpls.2018.01136 (PMC6085589; doi:10.3389/fpls.2018.01136)
Supplement: Supplementary file 2 [file Table_2.docx]

**Table S2** Information for allele-specific markers developed from known genes influencing flour color-related traits

| Gene | Chromosome | Marker | Allele | Fragment size (bp) | Phenotype |
| --- | --- | --- | --- | --- | --- |
| Phytoene synthase | 7AL | *YP7A* | *Psy-A1a/Psy-A1c* | 194 | High^a^ |
|  |  |  | *Psy-A1b* | 213 | Low^a^ |
|  | 7BL | *YP7B* | *Psy-B1a* | 151 | High^a^ |
|  |  |  | *Psy-B1b* | 156 | Low^a^ |
| Phytoene desaturase | 4B | *YP4B-1* | *TaPds-B1b* | 562 | High^a^ |
|  |  | *YP4B-2* | *TaPds-B1a* | 382 | Low^a^ |
| Lycopene ε-cyclase | 3A | *e-LCY3A-3* | *e-LCY3Aa* | 537 | - |
|  |  |  | *e-LCY3Ab* | 309 & 230 |  |
|  | 3B | *YP3B-1* | *Ta**Lcye-B1a* | 635 | - |
|  |  |  | *TaLcye-B1b* | No |  |
| 1B•1R translocation | 1B•1R | *H20* | Present | 1598 | High^a^ |
|  |  |  | Absent | No | Low^a^ |
| Lipoxygenase | 4BS | *LOX16* | *Lox-B1a* | 489 | High^b^ |
|  |  | *LOX18* | *Lox-B1b* | 791 | Low^b^ |
| Puroindoline b | 5D | *Pinb-D1b1/2* | *Pinb-D1a*  *Pinb-D1b* | No  250 | - |

^a^ Yellow pigment content, high or low following previous studies

^b^ Enzyme activity, high or low following previous studies

- Unknown
