# Supplementary material for: A Genome-Wide Association Study Reveals a Rich Genetic Architecture of Flour Color-Related Traits in Bread Wheat
Source: Front Plant Sci. 2018 Aug 3;9:1136. doi: 10.3389/fpls.2018.01136 (PMC6085589; doi:10.3389/fpls.2018.01136)
Supplement: Supplementary file 3 [file Table_3.docx]

**Table S3** Analysis of variance for flour color-related traits in 166 bread wheat cultivars

|  |  |  | L* | |  | a* | |  | b* | |  | YPC | |  |
| --- | --- | --- | --- | --- | --- | --- | --- | --- | --- | --- | --- | --- | --- | --- |
| Source of variation | *Df* |  | SS | *F* |  | SS | *F* |  | SS | *F* |  | SS | *F* |  |
| Environment | 3 |  | 88.94 | 301.60^**^ |  | 7.33 | 404.07^**^ |  | 176.93 | 570.18^**^ |  | 6.16 | 306.80^**^ |  |
| Replicate | 4 |  | 2.32 | 5.89^**^ |  | 0.11 | 4.40^*^ |  | 2.84 | 6.87^**^ |  | 0.47 | 17.47^**^ |  |
| Genotype | 165 |  | 1215.60 | 74.95^**^ |  | 156.56 | 156.92^**^ |  | 5414.11 | 317.23^**^ |  | 235.33 | 212.97^**^ |  |
| Genotype × Environment | 495 |  | 196.77 | 4.04^**^ |  | 13.97 | 4.67^**^ |  | 378.18 | 7.39^**^ |  | 6.91 | 2.09^**^ |  |
| Error | 636 |  | 62.51 |  |  | 3.85 |  |  | 65.79 |  |  | 4.26 |  |  |

L*, flour brightness; a*, flour redness; b*, flour yellowness; YPC, yellow pigment content

^*^ and ^**^ significant at *P* =0.001 and *P* =0.0001, respectively
