# Supplementary material for: A Genome-Wide Association Study Reveals a Rich Genetic Architecture of Flour Color-Related Traits in Bread Wheat
Source: Front Plant Sci. 2018 Aug 3;9:1136. doi: 10.3389/fpls.2018.01136 (PMC6085589; doi:10.3389/fpls.2018.01136)
Supplement: Supplementary file 4 [file Table_4.docx]

**Table S4** Pearson’s correlation coefficient among flour color-related traits based on BLUP values

| Trait | L* | a* | b* |
| --- | --- | --- | --- |
| a* | −0.01 |  |  |
| b* | −0.71** | −0.68* |  |
| YPC | −0.28* | −0.89** | 0.83** |

L*, flour brightness; a*, flour redness; b*, flour yellowness; YPC, yellow pigment content; BLUP, best linear unbiased predictor

* and ** significant at *P* =0.001 and *P* =0.0001, respectively
