# Supplementary material for: A Genome-Wide Association Study Reveals a Rich Genetic Architecture of Flour Color-Related Traits in Bread Wheat
Source: Front Plant Sci. 2018 Aug 3;9:1136. doi: 10.3389/fpls.2018.01136 (PMC6085589; doi:10.3389/fpls.2018.01136)
Supplement: Supplementary file 7 [file Image_1.PDF]

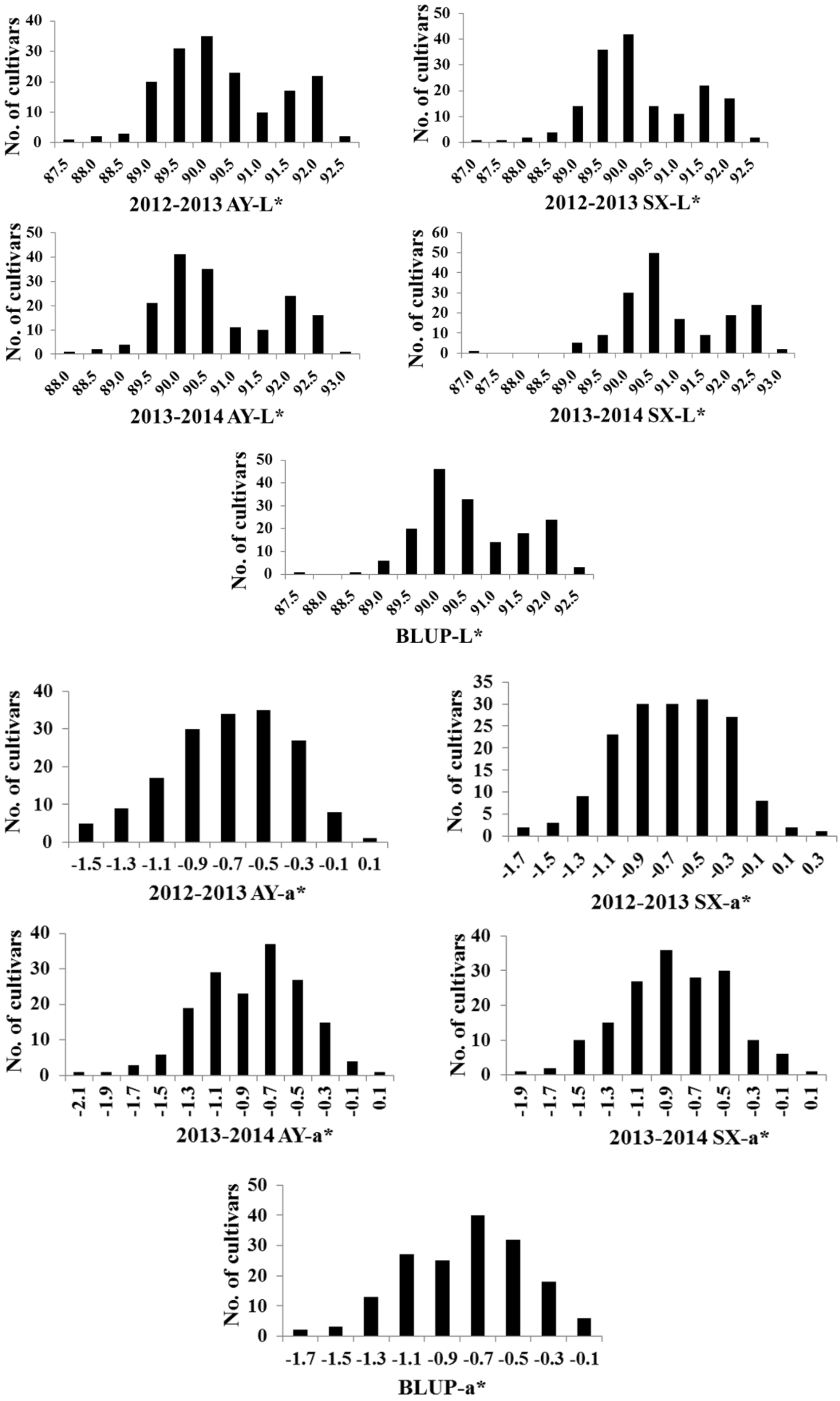

Figure S1 Frequency distribution of flour color-related traits in 166 bread wheat cultivars across four environments. L*, flour brightness; a*, flour redness; b*, flour yellowness; YPC, yellow pigment content (μg.g^{-1}); AY, Anyang; SX, Suixi; BLUP, best linear unbiased predictor.

Continued

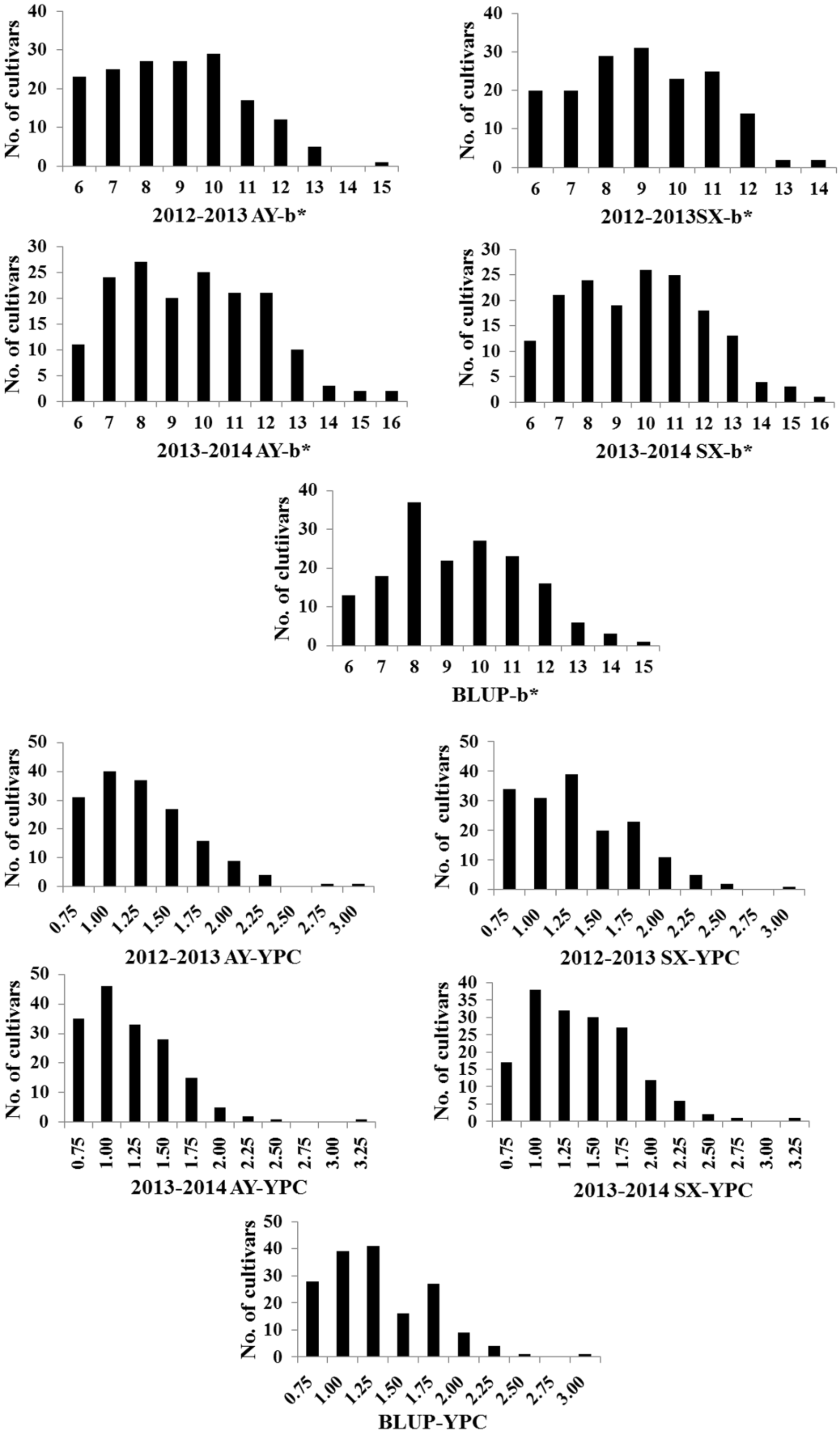
No. of cultivars
2012-2013 AY-b*
No. of cultivars
2012-2013SX-b*
No. of cultivars
2013-2014 AY-b*
No. of cultivars
2013-2014 SX-b*
No. of clutiivars
BLUP-b*
No. of cultivars
2012-2013 AY-YPC
No. of cultivars
2012-2013 SX-YPC
No. of cultivars
2013-2014 AY-YPC
No. of cultivars
2013-2014 SX-YPC
No. of cultivars
BLUP-YPC
