# Supplementary material for: A Genome-Wide Association Study Reveals a Rich Genetic Architecture of Flour Color-Related Traits in Bread Wheat
Source: Front Plant Sci. 2018 Aug 3;9:1136. doi: 10.3389/fpls.2018.01136 (PMC6085589; doi:10.3389/fpls.2018.01136)
Supplement: Supplementary file 8 [file Image_2.PDF]

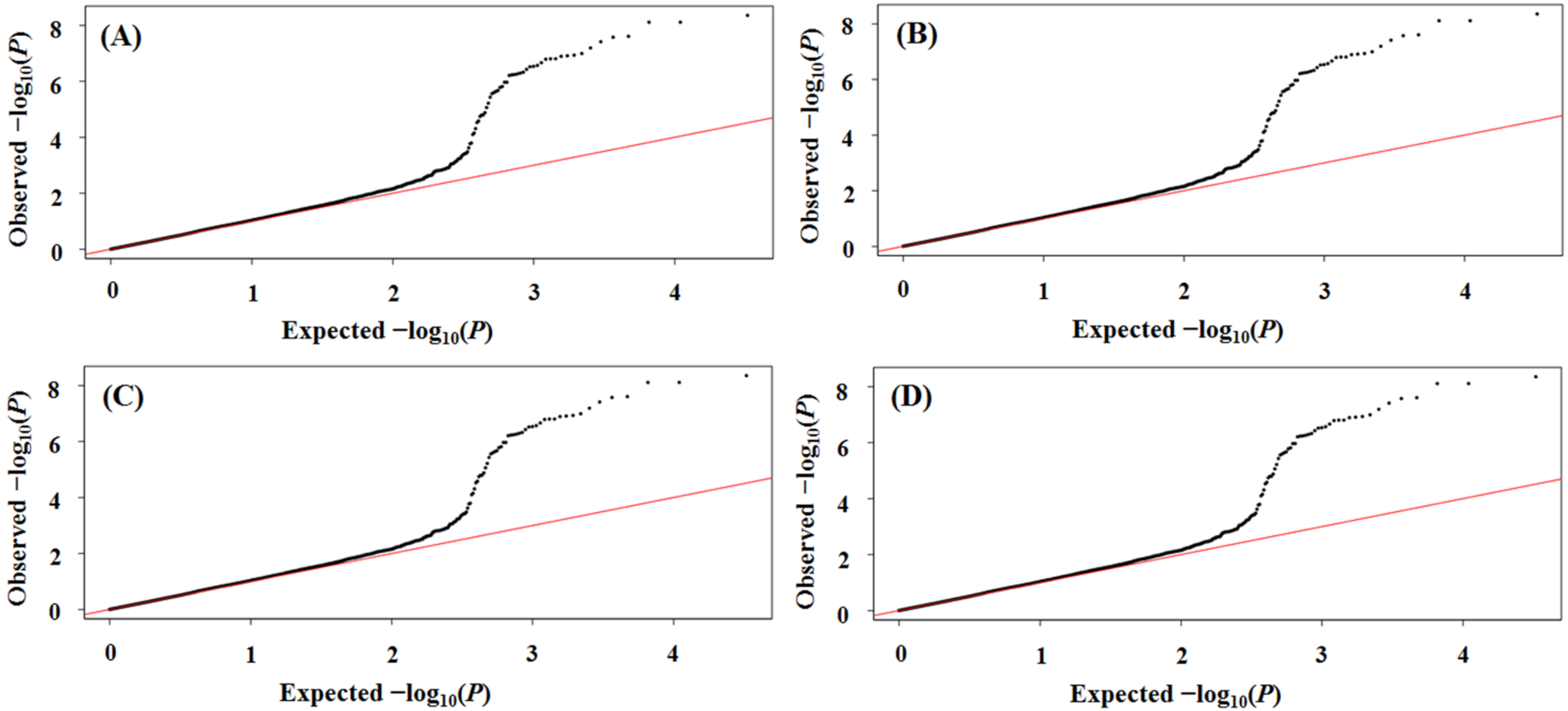

Figure S2 Quantile-quantile (Q-Q) plots of estimated $-\log_{10}$ (*P*). Q-Q plots of marker-trait association analysis for flour color-related traits based on BLUP values: (A) L*, (B) a*, (C) b* and (D) yellow pigment content (YPC). The red line is the expected line under a null distribution. In the upper right section of the graphs, deviations from the diagonal are expected, as these are the SNP most likely to be associated with the trait.
