# Supplementary material for: A Genome-Wide Association Study Reveals a Rich Genetic Architecture of Flour Color-Related Traits in Bread Wheat
Source: Front Plant Sci. 2018 Aug 3;9:1136. doi: 10.3389/fpls.2018.01136 (PMC6085589; doi:10.3389/fpls.2018.01136)
Supplement: Supplementary file 9 [file Image_3.PDF]

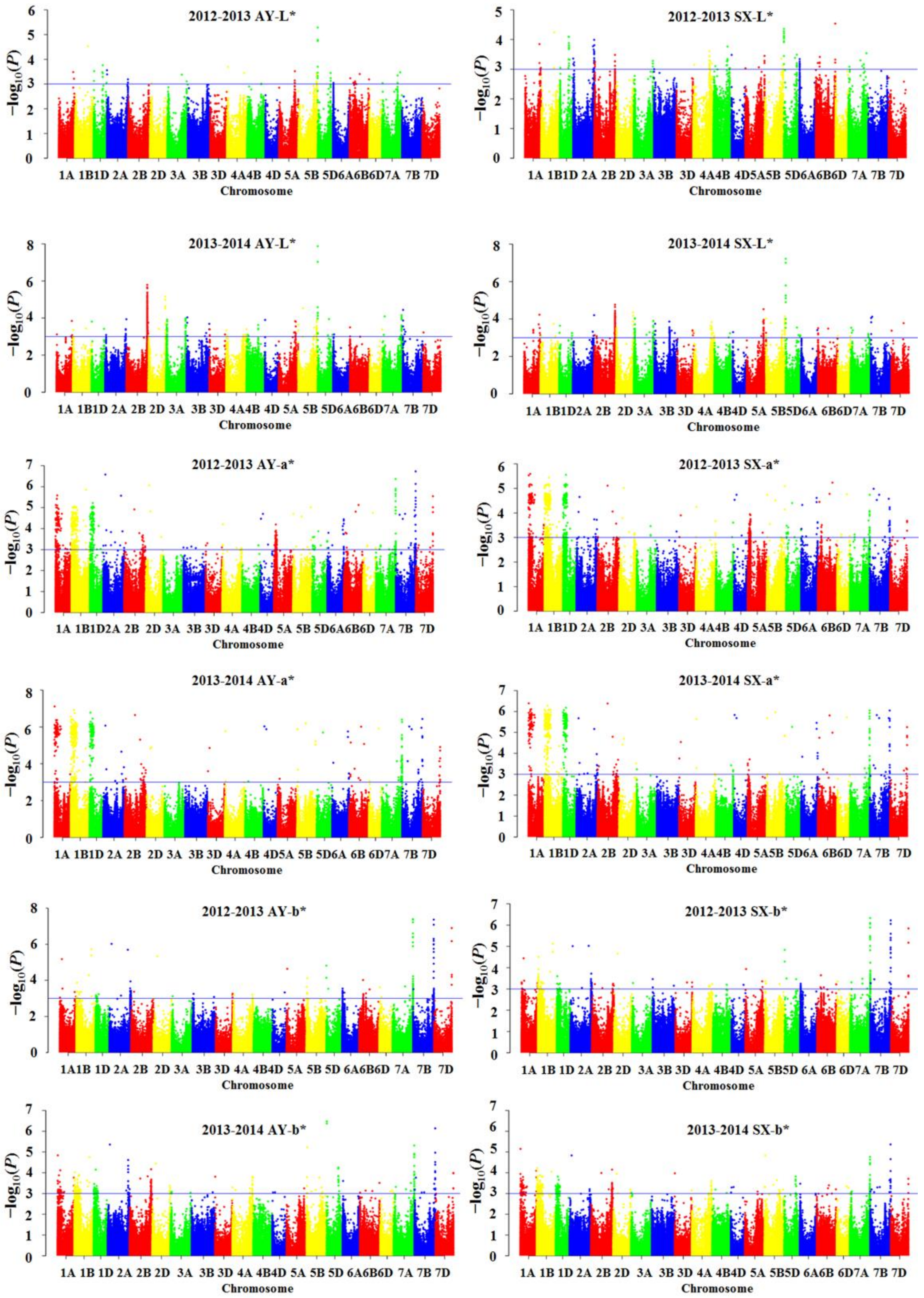

Figure S3 Manhattan plots indicating genomic regions associated with flour color-related traits in each environment. Negative $\log_{10}$-transformed *P* values from a genome-wide scan are plotted against SNP marker position on each wheat chromosome. The blue horizontal line designates the significant association threshold ($-\log_{10}(P) \geq 3$). AY, Anyang, SX, Suixi.

Continued

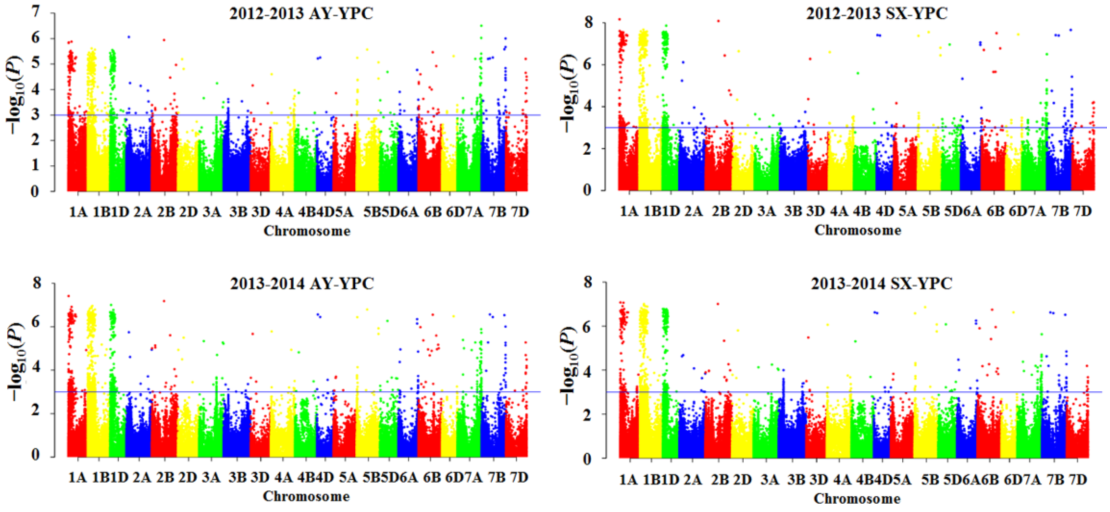
2012-2013 AY-YPC
−log10(P)
0 1 2 3 4 5 6 7
1A 1B1D 2A 2B 2D 3A 3B 3D 4A 4B4D5A 5B5D6A 6B 6D7A 7B 7D
Chromosome
2012-2013 SX-YPC
−log10(P)
0 2 4 6 8
1A 1B1D 2A 2B 2D 3A 3B 3D 4A 4B 4D 5A 5B 5D6A 6B 6D7A 7B 7D
Chromosome
2013-2014 AY-YPC
−log10(P)
0 2 4 6 8
1A 1B1D 2A 2B 2D 3A 3B 3D 4A 4B4D 5A 5B5D6A 6B 6D7A 7B 7D
Chromosome
2013-2014 SX-YPC
−log10(P)
0 2 4 6 8
1A 1B1D 2A 2B 2D 3A 3B 3D 4A 4B4D5A 5B 5D6A6B 6D7A 7B 7D
Chromosome
